# The Impact of Peri-Implant Diseases on the General Status of Patients with Cardiovascular Diseases: A Literature Review

**DOI:** 10.3390/life14060665

**Published:** 2024-05-23

**Authors:** Ana Maria Hofer, Alexandra Dadarlat-Pop, Alexandru Mester, Bogdana Adriana Nasui, Monica Popa, Andrei Picos

**Affiliations:** 1 Department of Community Health, “Iuliu Hatieganu” University of Medicine and Pharmacy, 400349 Cluj-Napoca, Romania; hoferana2000@gmail.com (A.M.H.); adriana.nasui@umfcluj.ro (B.A.N.); dr_monica_popa@yahoo.com (M.P.); 2 Cardiology Department, Heart Institute “N. Stăncioiu”, 400001 Cluj-Napoca, Romania; dadarlat.alexandra@yahoo.ro; 3 Department of Oral Health, “Iuliu Hatieganu” University of Medicine and Pharmacy, 400012 Cluj-Napoca, Romania; 4 Department of Oral Prevention, “Iuliu Hatieganu” University of Medicine and Pharmacy, 400012 Cluj-Napoca, Romania

**Keywords:** cardiovascular disease, peri-implantitis, coronary heart disease, dental implants

## Abstract

Background and Objectives: The aim of this study is to connect peri-implantitis to cardiovascular diseases, following the association found between periodontitis and cardiovascular conditions in recent years. Materials and Methods: PubMed, Scopus, Web of Science online databases were searched up to June 2023, with the exclusion criteria being research written in a language other than English. The MeSH search items were as follows: [“peri-implant health OR peri-implantitis OR peri-implant mucositis OR peri-implant disease”] AND [“cardiovascular diseases”]. Patient/population (P), intervention (I), comparison (C), outcome (O) framework questions were followed to identify the clinical evidence for the systematic review. Only clinical studies that used a control group to compare the relationship between cardiovascular diseases and peri-implantitis were selected. Results: A total of 118 studies were identified through electronic search of the keywords. After removing duplicates, there were 76 records to be screened. Upon exclusion of ineligible titles and abstracts, 27 studies remained for evaluation. Finally, 23 studies were excluded for not meeting the inclusion criteria, leaving 4 studies to be included in the qualitative analyses. Conclusions: This study found there is a linear association between mucosal/gingival inflammation and carotid intima–media thickness test (c-IMT) values. Peri-implant mucosal inflammation could be a contributor to the vascular disease burden of an individual; further specific clinical studies should be performed in order to demonstrate this connection.

## 1. Introduction

Drawing an analogy with the pathological conditions that affect the supporting tissues of teeth, gingivitis or periodontitis, the inflammation and destruction of soft and hard tissues around an implant are called mucositis and peri-implantitis [1].

Dental implants require time for osseointegration to take place; after that, biological conditions of infectious inflammatory origin can occur and affect the peri-implant tissue. This inflammatory process that occurs in implants is similar to that developed in natural teeth; infections have a lower resistance to destruction, mainly due to the lack of the periodontal ligament [1].

Peri-implant health has been defined both clinically and histologically [2,3]. Clinically, peri-implant health is characterized by the absence of visual signs of inflammation and bleeding on probing (BOP). Peri-implant health can exist around implants with normal or reduced bone support. This is clinically assessed by (2018 classification Araujo & Lindhe, 2018; Berglundh et al., 2018) [2,4]:

- Absence of inflammatory signs;
- Absence of bleeding on probing or suppuration;
- Stable probing depth between visits;
- Absence of bone loss beyond crestal bone level changes resulting from initial bone remodeling [2].

Mucositis describes a reversible inflammatory process, of bacterial origin, of the peri-implant tissues, characterized by the presence of inflammatory signs such as redness, edema and bleeding on probing. These clinical signs may not always be easy to identify, so bleeding on probing (BOP) remains an indicator of peri-implantation conditions [5].

Peri-implantitis is defined as “A pathological condition occurring in the tissues surrounding dental implants, characterized by inflammation in the peri-implant connective tissue and progressive loss of supporting bone” [3,6]. Unlike mucositis, peri-implantitis is a progressive and irreversible inflammation that affects both soft and hard tissues, being accompanied by bone resorption, lack of osseointegration, and the formation of purulent periodontal pockets [4,7,8,9,10]. Bleeding on probing, bone loss and deep probing depth can have reasons other than inflammation, such as deep insertion of the implant [11,12]. Furthermore, the type and shape of the implant, the type of connection, the material of the abutment and suprastructure, and the type of prosthetic suprastructure affects the peri-implant soft and hard tissues [12,13,14].

Peri-implant diseases are caused by bacterial biofilms and are associated with specific risk factors (Heitz-Mayfield & Salvi, 2018; Schwarz et al., 2018) [15,16]. The biofilm of the oral cavity contains a wide variety of bacteria. A spectrum of pathogens such as *Prevotella intermedia*, *Streptococcus constellatus*, *Aggregatibacter actinomycetemcomitans*, *Porphyromonas gingivalis*, *Treponema denticola* and *Tannerella forsythia* may be detected [17,18,19,20]. Because of this assortment of bacteria, peri-implantitis is considered a poly-microbial anaerobic infection [21]. However, unlike periodontitis, peri-implant lesions contain bacteria that are not the typical periodontopathogenic microbiota. In particular, Staphylococcus aureus seems to have a predominant role in the development of peri-implantitis. This bacterium shows a high affinity for titanium and has, according to the results of Salvi et al. a high positive (80%) and negative (90%) predictive value [22]. As another beneficial cause, smooth implant surfaces compared with rough surfaces may accelerate peri-implant inflammation [22,23,24].

Derks et al. showed a prevalence of peri-implant mucositis of 10% to 65% and peri-implantitis from 1% to 47% [25]. In contrast, Lee et al. showed a mean prevalence of peri-implantitis of 9.25% and peri-implant mucositis of 29.48% [26]. Such variations in prevalence are due to methodological heterogeneity in reporting peri-implant diseases, which limits the possibility of assessing the true impact of peri-implantitis globally [27,28].

Annually, the American Heart Association (AHA) publishes current statistics related to heart disease, stroke, and the cardiovascular risk factors [29,30]. The statistics updated in 2020 showed that the prevalence of total cardiovascular diseases (coronary heart disease, heart failure, stroke and hypertension) in adults (over 20 years of age) is 48% [29].

Chronic oral infections such as dental caries, periodontal disease or peri-implantitis are among the most common chronic inflammatory diseases [31]. The World Workshop on the Classification of Periodontal and Peri-Implant Diseases produced evidence that supports an association existing between severe periodontitis and chronic diseases such as diabetes [32,33,34,35] or chronic obstructive pulmonary disease [36,37]. Severe periodontitis appears to be a modifiable risk factor for CVD [38,39], significantly associated with all-cause and cardiovascular mortality [36], particularly among populations with multimorbidity [40,41].

In the present study, we considered the hypothesis that inflammation at affected peri-implant sites may induce low-grade systemic inflammation and increase the risk of cardiovascular disease through a potential infectious axis between the two diseases similar to that between periodontitis and CVD. The purpose of this study is to assess the relationship between peri-implantitis and cardiovascular disease (CVD) and oral disease on general health.

## 2. Materials and Methods

The PubMed, Scopus, and Web of Science online databases were searched up to June 2023, the exclusion criteria being written in a language other than English. The MeSH search items were as follows: [“peri-implant health OR peri-implantitis OR peri-implant mucositis OR peri-implant disease”] AND [“cardiovascular diseases”].

Once the articles were obtained, their selection was carried out according to an algorithm organized in two stages. The first stage involved evaluating the title and abstract of articles to achieve a reduction in the initial number of articles retrieved from the search and eliminate duplicates. There were three exclusion criteria for the articles represented by:

- Articles published in the last 10 years;
- The absence of the full text,
- Non-compliance with language restrictions.

After the selection of potentially eligible articles, the second stage involved obtaining the full texts of potentially eligible articles and their final evaluation. This was carried out using four inclusion criteria:

- The presence of the summary in the database;
- The presence of the main theme;
- The presence of the IMRAD-type structure (introduction, material and method, results, discussions);
- The existence of references for the article in question.

Our aim was to identify the existing scientific evidence from the current studies concerning the relationship between peri-implantitis and cardiovascular disease (CVD) by conducting a systematic review.

The focused questions to be addressed were the following:

(1) Are CVD and peri-implant diseases associated?
(2) If yes, what is the strength of evidence for an association between CVD and peri-implantitis compared to other potential risk factors?
(3) In patients participating in the studies with implants without peri-implant diseases, do those with CVD develop more peri-implantitis than those not suffering from CVD?
(4) If yes, is the prevalence of peri-implant diseases in participants associated with CVD?

Our protocol PICO (P = population, I = intervention, C = comparison, O = outcome) measures were the following:

P—population: participants with implants;
I—intervention: implant-related diseases (peri-implant mucositis or peri-implantitis);
C—comparison: subjects without comorbidities;
O—outcome: CVD (cardiovascular disease)

The population of interest were completely or partially edentulous patients restored by dental implants placed in mandibular or maxillary arches.

In order to investigate the association between peri-implantitis and CVD, we used the term CVD as exposure to capture cardiac diseases at any level and the underlying cause of diagnosis because of the difficulty to quantify the risks (such as diet, obesity, smoking, alcohol consumption, metabolic syndrome) and severity of cardiovascular pathology.

We included only studies that use a control group to compare the relationship between CVD and peri-implantitis without any host-factors (smoking, diabetes, immune susceptibility).

The outcomes were the presence or development of implant-related complications, such as peri-implant mucositis or peri-implantitis. In order to standardize the case definitions of peri-implant conditions, we used the ones proposed by the World Workshop on the Classification of Periodontal and Peri-Implant Diseases and Conditions and reported on behalf of its committee from the AAP and EFP [42], hereafter referred to as the European peri-implant disease case definitions.

Two reviewers conducted the selection process. The title and abstract selection were identified through the database search. The full-text reading was performed and the articles were considered eligible based on the inclusion/exclusion criteria.

## 3. Results

### 3.1. Study Selection

**Figure 1:**
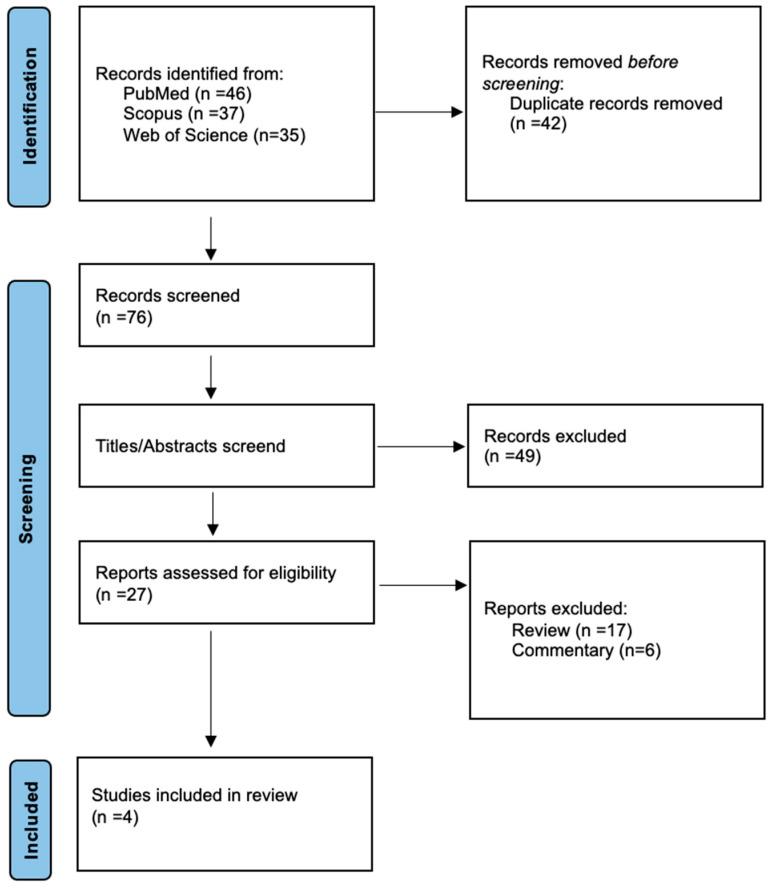
Study selection flow-chart.

A total of 118 records were identified through electronic search. After removal of the duplicates, there were 76 records to be screened. Upon exclusion of ineligible titles and abstracts, 27 studies remained for evaluation. Finally, 23 studies were excluded for not meeting the inclusion criteria, leaving 4 studies to be included in the qualitative analysis [43,44,45,46] (Figure 1).

### 3.2. Study Characteristics

Each of the four studies included a minimum of 58 peri-implantitis subjects, with comorbidities potentially affecting their periodontal/peri-implant status.

Blood sampling and analysis of serum concentrations of triglycerides, total cholesterol, LDL, HDL and vitamin-D, uric acid, plateletcrit, neutrophil, hemoglobin were measured in two studies [43,44]. One cross-sectional study investigated the association between carotid intima–media thickness (c-IMT) values and periodontal and peri-implant diseases.

Clinical periodontal parameters around natural teeth were reported in all selected studies. Clinical attachment level (CAL) and periodontal probing depth (PPD) were both measured in four studies, just as the bleeding on probing (BOP). Gingival index (GI) and plaque index (BI) were assessed in two studies [45,46]. Only one study evaluated other periodontal parameters, such as keratinized mucosa width (KMW). Regarding radiographic bone loss, three studies included it as a parameter around the implant.

Of the four clinical studies included in the present systematic review, two were cross-sectional, one was a retrospective and one was a case-control study. No longitudinal nor randomized clinical trials met the eligibility criteria. A complete description of the selected studies, regarding participants, intervention, comparison and outcomes and considerations and conclusions focusing on the PICO questions is reported in Table 1.

The results found in the literature concerning the relationship between risk markers for cardiovascular disease and peri-implant diseases focused on the coexistence of medical conditions. Evidence suggests that bone tissues may alter as a result of high levels of triglycerides and cholesterol as well as cardiovascular diseases. No statistically significant difference was identified between groups in terms of LDL-C, HDL-C and TOTAL-C values. Positive correlation was found between uric acid, triglycerides and GI, PD, BOP and KMW values [43].

The cardiovascular group had a significantly higher prevalence of moderate to severe peri-implantitis [47].

The subject analyzed in this paper is a relatively new topic; therefore, the number of studies found is very limited; also, the number of patients is reduced. Because of this, we are reticent in presenting to the patient the impact of peri-implantitis on cardiovascular disease. To continue this research path, we need to begin a clinical study with hopefully many more patients included than in the study before us, in order to consolidate the peri-cardio relation.

## 4. Discussion

The aim of the study was to demonstrate the impact of peri-implantitis on general health. Different systematic reviews of observational studies [48,49] showed how patients with periodontitis have higher carotid intima–media thickness (c-IMT) values.

The present systematic review, unlike the previous ones [48,49] including studies on the risk markers of cardiovascular diseases, aimed to primarily evaluate the correlation of factors affecting peri-implant bone resorption. The periodontal parameters around natural teeth and dental implants were used in all of the included studies [43,44,45,46]. The included studies’ CVD group had a significantly higher prevalence of moderate to severe peri-implantitis (RBL ≥ 2 mm) [46].

The clinical periodontal parameters around implants were recorded in all selected studies; however, there were only two studies describing the calibration process conducted by the researcher [44,45], a factor which increases the accuracy of the dental examinations. Similarly, heterogeneous peri-implant health, mucositis and peri-implantitis case definitions were applied in the included studies, different from the one introduced by the 2018 classification of periodontal and peri-implant diseases and conditions [42].

In Sanz et al., 2020 [50] consensus report, the term CVD is used as a general term for atherosclerotic diseases, principally coronary heart disease, cerebrovascular disease and peripheral vascular disease. There are many chronic infections, inflammatory and immune diseases that have a higher risk of cardiovascular events, such as arthritis, systemic lupus erythematosus or periodontitis [51,52,53,54]. The major common risk factors for CVD remain the lifestyle factors, principally tobacco smoking, dyslipidemia, hypertension and altered glucose metabolism, which also represent the risk factors for periodontitis [53,55,56,57].

Although peri-implantitis mainly shares etiopathogenic pathways with periodontitis [43], any certain association between peri-implant disease and cardiovascular diseases has not been established. In the works analyzed, most of the studies that analyzed CRP (C-reactive protein) levels after the periodontal treatment showed a statistically significant reduction in this parameter [50,58]. The periodontal treatment applied was the same in all studies, but the different outcomes could be explained by the fact that patients with gingivitis were included, rather than severe chronic periodontitis, and that baseline values were significantly different between the intervention and control groups [59,60].

In Chu D. et al., 2023 [61], a recent meta-analysis selected studies that included patients with cardiovascular diseases and estimated the risk of potential peri-implant diseases in these patients. The study concluded that there is a high incidence of peri-implantitis in patients suffering from cardiovascular diseases. This demonstrates a circular relationship between these pathologies [62,63].

Similar to periodontitis, in peri-implantitis, the crevicular fluid shows an increase in pro-inflammatory cytokines (IL-6, IL-10), but the vascular structure of the infiltrated connective tissue is more dense [64,65,66]. This suggests that peri-implantitis lesion has a different histopathology and can show the rapid progression which may plausibly have systemic effects [67].

This evaluation highlights the need for new research approaches regarding the impact of peri-implant diseases on cardiovascular pathology. With this in mind, a new clinical study is to be completed, in order to demonstrate this bidirectional connection. For this study to be validated, the values of inflammatory markers such as lipoprotein-associated phospholipase (Lp-PLA2), metaloproteinase-8 (MMP-8), myeloperoxidase (MPO) and soluble CD40-ligand have to be examined before and after the periodontal treatment.

The preventive measures we think should be performed in order not to develop cardiovascular complications are not the analysis of c-IMT values, but professional modern periodontal treatment on implants diagnosed clinically and radiologically with peri-implantitis, which is also the golden standard treatment.

Specific periodontal treatments are mandatory in patients with or without cardiologic pathologies and are low in cost compared to cardiological investigations; therefore, the approach should be in this order; if the first approach is the periodontal treatment, then the c-IMT values will decrease, because we conclude that peri-implantitis has the same bacterial involvement as in dental periodontal pockets [49,68].

## 5. Conclusions

In conclusion, the results found in the periodontal parameters, and the current lack of such data related to peri-implant tissues and to alveolar bone loss highlight the need for further studies on the topic, potentially paving the way for a more comprehensive approach to periodontitis and peri-implantitis management. Indeed, there is a linear association between mucosal/gingival inflammation, inflammatory profile and c-IMT values.

Peri-implant mucosal inflammation could be a contributor to the vascular disease burden of an individual. Specific periodontal treatment needs to be applied when the first sign of peri-implant inflammation appears; for interventions on the implant, special dental instruments are required in order not to damage the implant surface: plastic and titanium instruments, also glycine air–powder abrasion instead of calcium-carbonate, due to the smaller dimensions of the particles. Periodical oral and radiographic examinations need to be performed to ensure primary and secondary prophylaxis.

## Figures and Tables

**Table 1:** Associations between cardiovascular diseases and peri-implant diseases.

Included Studies	Methods	Periodontal Paramters Evaluated	Outcome(s)	Conclusions
Author Year Study design	Participants Subjects (n.) Intervention CVD Comparison CVD/Non-CVD Procedure(s) Any	Clinical CAL PPD BOP Gingival index(GI) Plaque index(PI) Tooth loss Radiographic Bone loss(RBL) Biochemical parameters Inflammatory mediators	Statistically significant (*p* < 0.05)	
Gülbahar Ustaoğlu 2020 Cross-sectional study [43]	Participants: Peri-implantitis 58 Peri-implant mucositis 49 periodontal health 49 Intervention: Non-CVD Comparison Serum biochemical parameters in selected subjects Procedure(s): - Periodontal exam - Blood sampling and analysis of serum triglycerides, total cholesterol, LDL, HDL and vitamin-D, uric acid, plateletcrit, neutrophil, hemoglobin	Clinical CAL PD PI GI BOP KMW	Uric acid and Gi, PD, BOP, KMW correlation (r = 0.238, *p* = 0.006; r = 0.0464, *p* ≤ 0.001; r = 0.230, *p* = 0.008; r = 0.240, *p* = 0.006) Vitamin-D and GI (r = −0.191, *p* = 0.020)	Higher levels of triglyceride and uric acid (risk markers for a cardiovascular disease) in peri-implantitis group. No statistically significant differences were found between the groups in terms of LDL-C, HDL-C and TOTAL-C values. Positive correlation between uric acid, triglyceride and GI, PD, BOP and KMW values.
Piero Papi 2022 Cross-sectional study [44]	Participants 151 participants with at least one dental implant in function for >5 years Intervention CVD patients Comparison c-IMT and presence of plaque Procedure(s) - Periodontal exam - Ultrasound assessment of carotid arteries - Venous blood sampling (fasting plasma glucose, total cholesterol, HDL-C, LDL-C, triglycerides, high-sensitivity C-reactive protein, creatinine, uric acid)	Clinical CAL PPD(mm) PI GI BOP Radiographic Bone loss	GI (β = 0.011, SE 0.002, *p* < 0.001) CAL (β = 0.114, SE 0.020, *p* < 0.001) peri-implant diseases (β = 0.011, SE 0.002, *p* < 0.001) with increased c-IMT values	A linear association between mucosal/gingival inflammation and c-IMT values. Peri-implant mucosal inflammation could be a contributor to the vascular disease burden of an individual.
Stefan Renvert 2013 Retrospective study [45]	Participants Peri-implantitis 172 Peri-implant health/mucositis 98 Intervention CVD patients Comparison CVD patients/non-CVD Procedure(s) - Periodontal exam - Medical history	Clinical CAL PPD (mm) BOP Radiographic Bone loss (>2 mm)	PDD: mean 5.5 mm (SD ± 0.8) and 4.2 mm (SD ± 1.1) with a mean difference of 1.2 mm (SE ± 0.2 mm, 95% CI: 3.1, 3.9, *p* < 0.001), *p*-value = 0.001. Bone level 5.0 mm (SD ± 1.7) and 1.5 mm (SD ± 0.4), mean difference of 3.5 mm (SE ± 0.2, 95% CI: 3.1, 3.8, *p* < 0.001) History of CVD (%) (peri-implantitis 27.3, Implant health/mucositis 3.0, *p*-value = 0.001)	In relation to a diagnosis of peri-implantitis, a high likelihood of comorbidity was expressed by a history of periodontitis and a history of cardiovascular disease.
I-Chang Wang 2021 Case-control study [46]	Participants 128 participants (CVD group, *n* = 82, control group, *n* = 46) Intervention CVD patients Comparison CVD patients/non-CVD Procedure(s) - Periodontal exam - Medical history	Clinical CAL PPD(mm) BOP Radiographic Bone loss(<2 mm, 2 to 4 mm, >4 mm)	Peri-implantitis and CVD (odds ratio = 2.18, 95% CI, 1.02 to 4.67; *p* = 0.04) PPD (≥7 mm) and BOP +(>66%) when compared with controls (*p* > 0.05)	CVD group had significantly higher prevalence of moderate to severe peri-implantitis (RBL ≥ 2 mm).

## Data Availability

Data is contained within the article.
